# Development of a Digital Platform to Promote Mother and Child Health in Underserved Areas of a Lower-Middle-Income Country: Mixed Methods Formative Study

**DOI:** 10.2196/48213

**Published:** 2024-07-31

**Authors:** Zaeem Ul Haq, Ayesha Naeem, Durayya Zaeem, Mohina Sohail, Noor ul Ain Pervaiz

**Affiliations:** 1 Gavi, The Vaccine Alliance, Islamabad, Pakistan; 2 Health Services Academy, Islamabad, Pakistan; 3 Onto Global, Islamabad, Pakistan; 4 Alliance for Behavioural Communication & Development, Islamabad, Pakistan

**Keywords:** primary health care, mother and child health, community health worker, slums, digital applications, health communication.

## Abstract

**Background:**

Primary health care (PHC) is the backbone of universal health coverage, with community health workers (CHWs) being one of its critical pillars in lower-middle-income countries. Most CHW functions require them to be an efficient communicator, but their program development has been deficient in this area. Can IT provide some solutions? Moreover, can some IT-based CHW-delivered innovations help mothers and children in areas not covered by PHC services? We explored these questions during the development and feasibility testing of a digital application designed to improve the communication capacity of CHWs in two underserved areas of Islamabad.

**Objective:**

This study aims to explore the perceptions, practices, and related gaps about mother and child health, and child development in an underserved area; develop and deploy a behavior change communication program to address the gaps; and assess the feasibility of the program.

**Methods:**

We carried out a mixed methods study with three steps. First, we conducted 13 in-depth interviews and two focus group discussions with stakeholders to explore the issues faced by mothers living in these underserved areas. To address these barriers, we developed Sehat Ghar, a video-based health education application to demonstrate practices mothers and families needed to adopt. Second, we trained 10 volunteer CHWs from the same community to deliver health education using the application and assessed their pre-post knowledge and skills. Third, these CHWs visited pregnant and lactating mothers in the community with random observation of their work by a supporting supervisor.

**Results:**

Initial exploration revealed a need for health-related knowledge among mothers and suboptimal utilization of public health care. Sehat Ghar used behavior change techniques, including knowledge transfer, enhancing mothers’ self-efficacy, and improving family involvement in mother and child care. Volunteer CHWs were identified from the community, who after the training, showed a significant improvement in mean knowledge score (before: mean 8.00, SD 1.49; after: mean 11.40, SD 1.43; *P*<.001) about health. During supportive supervision, these CHWs were rated as excellent in their interaction with mothers and excellent or very good in using the application. The CHW and her community reported their satisfaction with the application and wanted its delivery regularly.

**Conclusions:**

Sehat Ghar is a simple, easy-to-use digital application for CHWs and is acceptable to the community. Mothers appreciate the content and presentation and are ready to incorporate its messages into their daily practices. The real-world effectiveness of the innovation tested on 250 mother-infant pairs will be important for its proof of effectiveness. With its usefulness and adaptability, and the rapidly spreading use of mobile phones and internet technology, this cost-effective innovation can help in delivering health communications at a large scale in a minimum amount of time.

## Introduction

Primary health care (PHC) is the backbone of universal health coverage, which in turn, is critical in the agenda of Sustainable Development Goals [1]. COVID-19 also highlighted the significance of PHC—countries with strong PHC outbreak response systems were more successful in fighting the pandemic [2]. PHC has several essential elements, community health workers (CHWs) being one of them, especially in lower-middle-income countries. Acting as an extension of the health facility, these workers have linkages with families and are responsible for health education, essential medication, and referral for the cases that need to be assessed at the health facility [3].

The performance of these CHWs is crucial for the effectiveness of several dimensions of a health system [4,5]. For example, efficient delivery of health education improves community health behaviors that reduce disease burden and overall health expenditures. Early recognition of symptoms and provision of essential medication by CHWs at the household prevents a family’s need to visit their health facility, saving money and avoiding crowding at the facility [6]. Timely identification and referral of complicated cases save lives and improve health [7]. All these functions require the CHW to be an effective communicator. Evaluations of CHW programs, however, usually ignore this primary function [8,9].

Researchers have focused on using digital and mobile phone technology to improve CHW programs. Digital and mobile health interventions have been tested, including those for data collection, reminder SMS text messaging, online training, and delivery of video content on mobile phones [10]. The information gleaned from these studies is mixed. For example, a need for more theoretical models has been prominent in the discourse about interventions for behavior change [11,12]. Moreover, incomplete information from the formative and process evaluation of these studies makes their upscale a challenge. Some have also questioned the usefulness of these technology-based interventions because of the costs, dependence on internet availability, and the training requirements [13].

We explore the answer to these questions by developing and pilot-testing a CHW-delivered digital intervention for populations not covered by PHC in Pakistan. According to the most recent population census in 2023, about 39% of the population in Pakistan lives in urban areas, 56% of which is in underserved areas and squatter settlements [14,15]. These informal settlements usually fall outside the mainstream health and governance system, which is already stretched and unable to provide for even the well-settled populations [16,17]. Women and children face further health challenges because of gender intersectionality [18]. Biomedical interventions to address maternal and child survival and health requirements are available but with partial coverage [19,20].

In this paper, we report on the development and feasibility testing of Sehat Ghar (Urdu for “health house”), an Android-based digital application. Volunteer CHWs from the local area are using this application to promote mother and child health among the families living in 2 of the 34 squatter settlements of Islamabad, Pakistan. This formative study aims to pilot-test a tailored health communication intervention that fulfills the knowledge and skill-building needs of a community not covered by the PHC services. The findings will also help other researchers aiming to use digital technology to empower communities and strengthen PHC systems.

## Methods

### Setting, Design, and Study Objectives

**Figure 1.**
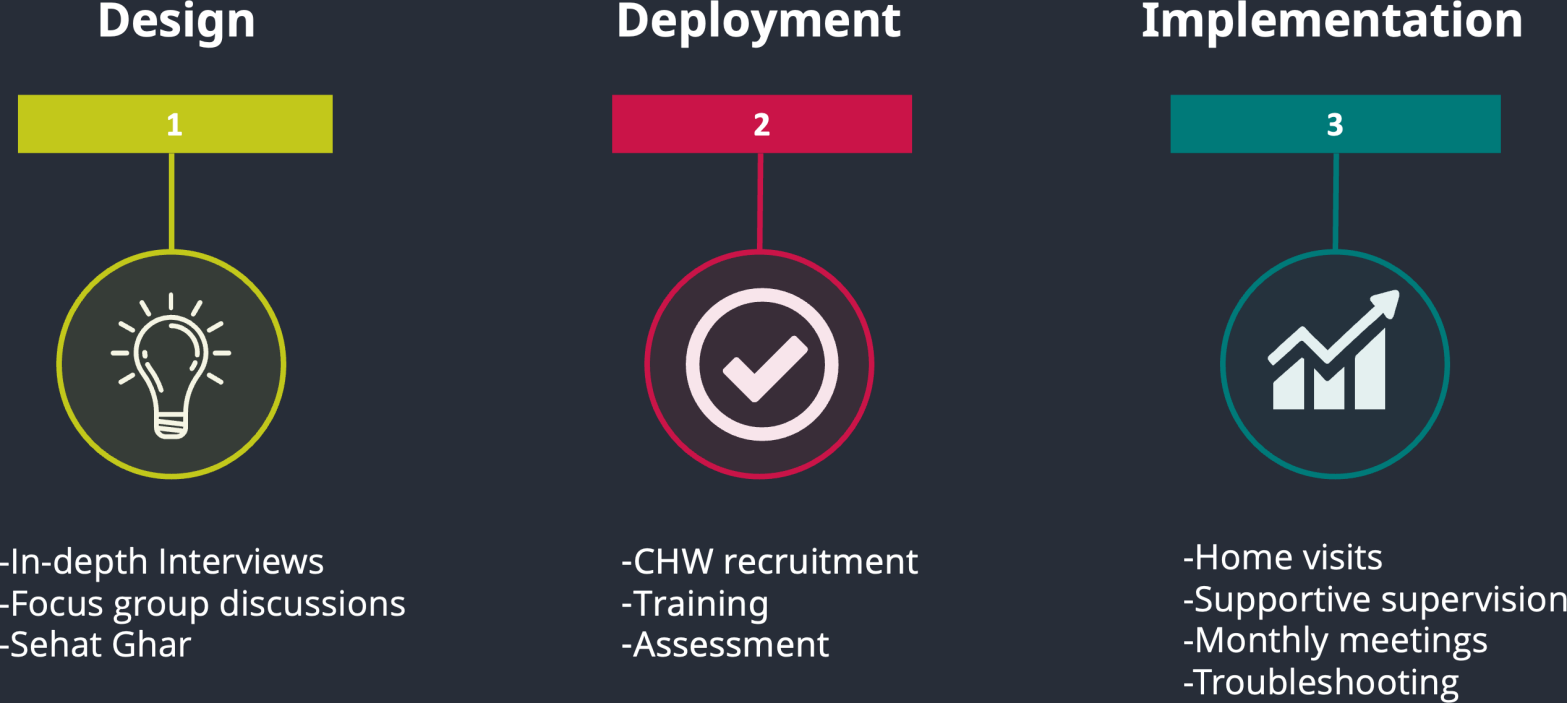
Three phases of Sehat Ghar: design, deployment, and implementation. CHW: community health worker.

We conducted a mixed methods participatory study (Figure 1) in line with our earlier community-based formative studies [21]. The study involved two underserved areas of Islamabad, Pakistan’s capital, with a geographical area of 906 km^2^ and a population of over two million [14]. The city has at least 34 underserved areas inhabited by 85,000 people [22]. Many of these underserved areas have poor water, sanitation, and hygiene conditions, and are devoid of public-funded PHC services [23]. We focused our study on France and the Rimsha colonies, two squatter settlements located close to the city’s center, with a population of 8000 and 10,000, respectively [24]. We selected these colonies because they fulfilled our criteria of a minimum population of 5000, having a distance of 3‐5 kilometers from a functioning public hospital where cases could be referred, and health care providers (HCPs) willing to support our study.

The residents of both areas mainly belong to the Christian (minority) faith and laborer class, and have low socioeconomic status and poor coverage of PHC services. Our preliminary visits to these areas revealed that there was no significant presence of CHWs from the public sector in these areas. People visited private health providers (both formal and informal) for treatment of minor ailments, and either of the two public hospitals (Polyclinic and Pakistan Institute of Medical Sciences) for a serious condition or hospitalization. Water-borne and other environment-related health problems were common, with mothers and children impacted the most in these areas [17,23].

The following were the specific objectives of this study:

- Explore the perceptions, practices, and related gaps about mother and child health, and child development
- Develop and deploy a behavior change communication program to address the gaps
- Assess the feasibility of the program

### Study Participants

We contacted all practicing HCPs in the area—formal and informal—and offered them involvement in our study as a stakeholder. Of the 9 HCPs, 5 (n=2 from Rimsha and n=3 from France colony) agreed. Through them, we identified volunteer women from the community willing to become a CHW. Women were preferred because they could easily visit the households and talk about issues like birth preparedness and complication readiness [25,26]. In addition to being female, 10 years of schooling, living in the same community, and preferably being married were the criteria for their selection. A total of 15 volunteer women were enrolled. Upon our request, these volunteers conducted household visits and enlisted pregnant or nursing mothers in their area. We invited 10 mothers (5 from each cluster from the list) for in-depth interviews (IDIs) as part of this formative study. Overall, we interviewed 5 HCPs and 8 mothers (2 did not agree to interview), and conducted two focus group discussions (FGDs) with 15 volunteer women (future CHWs for our study) during the inception phase.

### Data Collection

Data were collected during all three phases (Figure 1) of the study. Our overarching question was, “In an underserved area, how do pregnant women and children access healthcare, and what are the gaps at the household level that a behavior change intervention can address?” During phase 1, qualitative data were collected to explore the HCP views about the health-seeking practices of their community during the antenatal, natal, and postnatal periods. In the FGDs with future CHWs, we explored the community practices regarding mother and child health, child development, and the gender dimensions prevalent in that culture. Through the IDIs with mothers, we explored the family practices around mother and child nutrition, health-seeking, and child development.

During phase 2, CHWs received training and their knowledge was tested before and after their training, using a yes/no questionnaire. During phase 3, the CHWs visited mothers in their community; their engagement with mothers and families was observed and recorded. A checklist with 21 items (yes/no) was developed for this purpose to record observations on *fidelity to intervention* (6 items), *mastery of using the tablet* (5 items), and *command over the content* (10 items). All CHWs knew that a supervisor would visit them during their household sessions, but the date and time were not revealed to them. Toward the end of a visit, the supervisor also sat with the mother and explored her qualitative feedback using open-ended questions.

For qualitative discussions, two team members with extensive qualitative research experience conducted the interviews and FGDs in Urdu, a language convenient to the community and the study team. The interviews of HCPs and mothers were conducted at the clinic and at home, respectively, while the FGDs were conducted, with consent, in the courtyard of a CHW house. The IDIs took about 30‐50 minutes, and a typical FGD was 90 minutes. All discussions and interviews were tape-recorded, transcribed, and translated into English.

### Data Management and Analysis

For qualitative data, the team transcribed the Urdu discussions from interviews and FGDs and translated these into English. Two team members independently read and coded the initial five transcripts to develop a code list. This initial code list was discussed to see congruence between the two coders and resolve disagreements to reach a final, consensus code sheet. This final code sheet was then used for all interviews and discussions. The analysis identified significant statements across all transcripts using inductive techniques [27,28]. These meaning statements were clustered together to identify themes. We gave weightage to recurring themes; however, we also paid attention to the divergent themes or points that were not shared by many participants but appeared significant [29]. For quantitative analysis, we compared mean scores from the knowledge test of CHWs using a paired 1-tailed *t* test for the hypothesized increase in CHW knowledge after the training. The quantitative data from implementation monitoring were rank-ordered to make supportive supervision decisions during the implementation.

### Ethical Considerations

The study team consisted of researchers with medical, public health, and sociology backgrounds; training in mixed methods research; and experience in formative and process evaluations. Most of the team members were female, as the nature of work required close collaboration with mothers and other female members of their households. No incentives or payments were offered to the participants of this research at any stage. To ensure confidentiality and privacy, all data were deidentified for presentation. The Institutional Review Board of the Health Services Academy, Islamabad, Pakistan, approved the study (7‐82/IERC-HSA/2018‐06). Grand Challenges Canada funded this study through a seed grant (ST-POC-1808‐17445), and this formative phase was completed from April to August 2018.

### Intervention

In their discussions, the study participants identified knowledge gaps about mother and child health and child development. The discussions also revealed a lack of self-efficacy among mothers to organize their actions around better health outcomes. A digital platform was developed to address the knowledge gaps and provide a platform for the CHW to help the mother and her family through discussions. Applying human-centered design principles [30,31], a multidisciplinary team developed this intervention through several stages. To begin with, the team summarized the findings from stakeholder consultations in the form of a matrix (Table 1) to highlight the community’s existing knowledge and skills and relevant gaps. Priority behaviors were identified with the help of HCPs and CHWs, and health education content was finalized to address the gaps.

**Table 1.** Gaps in community practices and the intervention content to address the gaps.

Themes	Findings	Intervention element
Access to primary health care	No coverage of PHC^a^ at the household level Mothers unaware of healthful practices at household level Families trapped in poverty	Volunteer CHW^b^ introduced as substitute CHW received basic training to deliver the intervention Emphasis on behaviors that can prevent disease, promote health, and save costs
Mother’s health and nutrition	Lack of knowledge about antenatal and postnatal care, and regular checkups Lack of knowledge about maternal nutrition, infrequent use of iron tablets Mental stress during pregnancy	Sessions 1‐3 on antenatal and postnatal checkups, danger signs, and steps to be taken if danger signs appear Emphasis on maternal nutrition, especially iron/folate Family support appreciated and further emphasized especially to husband
Newborn health	Families opt for formal health facility, yet some also prefer TBA^c^ for childbirth Newborns receive checkup only if delivered at health facility Prelacteals are common; breastfeeding delayed for 1‐3 days Warm *desi ghee* (butter) applied to umbilical cord	Specific information about SBA^d^ and their importance Session 3 on newborn care: Significance of early checkup Identifying and taking action for danger signs in a newborn Early initiation of breastfeeding, delayed bathing for 24 hours Using chlorhexidine for cord care emphasized
Child health and nutrition	Children taken to hospital only for immunization or in case of emergency Lack of knowledge about duration and significance of exclusive breastfeeding Perception that breastmilk is insufficient Water and semisolids frequently used between 2‐5 months Lack of knowledge about child nutrition	Sessions 3‐7 focus on child health and encourage families to consult health care provider whenever necessary Session 5 and 6 specifically talk about preventable diseases, their symptoms, and child immunization Sessions 4‐7 emphasize exclusive breastfeeding for 6 months, and explain child’s nutritional requirements with growing age These sessions also include homemade recipes for adding semisolid diets for children older than 6 months
Environment for child’s development	No awareness and understanding of child stimulation and play Families focus on child nutrition and link nutrition only with physical growth	Sessions 4‐7 explain stimulation and play, and provide age-appropriate play activities for children Content on making toys from everyday items to enhance child stimulation and play

a PHC: primary health care.

b CHW: community health worker.

c TBA: traditional birth attendant.

d SBA: skilled birth attendant.

Participant mothers and CHWs preferred stories to convey health information. A creative team comprised of a graphic designer, a scriptwriter, and a video producer was engaged to record short docudramas to address the information gaps. Once the graphics and audio-visual clips were ready, we collaborated with the application development team to transfer the entire set into a user-friendly, easy-to-navigate digital package. Called Sehat Ghar, this digital application was installed on a tablet for the CHWs to use in their counseling sessions with the mothers. The cost of development was US $1000 for the 12 videos and US $3000 for the development of the Android application. Upon CHWs’ recommendation, a wall calendar for mothers was also developed that displayed the graphics and short messages from the video. In between the two visits, this calendar would remind the mother about the actions she needed to carry out for her and her baby. Input from CHWs and the community was incorporated into these pieces at all stages of the intervention development.

The Sehat Ghar intervention comprised 7 sessions that used 14 live-action videos and a health calendar (Table 1). Delivered by the CHW at the mother’s house at monthly intervals, the sessions started from the last trimester of pregnancy until the child was aged 6 months. The content included timely and appropriate messages on maternal health and nutrition, child health and nutrition, the importance of a skilled birth attendant, immunization, and a positive home environment. With this package, we theorized that CHW-delivered Sehat Ghar content would improve the knowledge and self-efficacy of mothers about the steps that can be ensured at home. Moreover, family practices, including facilitating the mother to go to a nearby public health facility for health care will also improve. The combined result of these practices will be an improvement in mother and child health, and child development.

### Deployment of the Program

A 2-day training was offered to all 15 CHWs enrolled in the study. Due to personal or family reasons, 5 dropped out at different time points, and a final set of 10 CHWs completed all steps of training and apprenticeship. The process comprised the main training followed by 1 week of apprentice work in the field and a 1-day training refresher after CHWs had completed apprenticeship in the field. The training curriculum comprised video-based health education content to address gaps in the knowledge about mother and child health, interpersonal communication, and skill development for using the tablet and the Sehat Ghar application.

The CHW-led sessions comprised two live-action videos for discussion. The videos presented a contrasting story through human characters. The first story showed a household where the family did not encourage a mother’s healthful nutrition, with the mother and her baby ultimately facing health consequences. The second story presented a family with a similar socioeconomic status but positive thinking and self-belief, dealing with economic problems yet ensuring healthy food for the mother and the baby. After watching the videos together, the CHW discussed similar problems the participant mother and family may be facing and the best ways to jointly solve them. Applying a simplified cognitive behavioral technique (CBT) used in our community-based studies earlier [21], the CHW discussed the practical activities that a mother and family could implement to solve those problems. In a nondidactic manner, the CHW would emphasize interspousal discussions for problem-solving and making the best use of available resources.

### Implementation and Testing

Once the CHW started home visits, a field supervisor (also involved in CHW training) regularly conducted field visits to observe the field performance and practices of CHWs. Using a 21-item (yes/no) checklist, the supervisor assessed the CHW’s engagement with the family. At the end of each visit, the supervisor appreciated a satisfactory CHW performance and discussed any areas of improvement. Data from the initial 20 visits (2 visits per CHW) were analyzed to carry out the performance ranking of CHWs and draw lessons for collective feedback. On the first follow-up after 3 days, qualitative feedback was also obtained from CHWs about the Sehat Ghar application and use of the digital tablet. In addition, the supervisor also obtained qualitative feedback from mothers about the usefulness of CHW visits and the content of the Sehat Ghar application.

## Results

A final cohort of 10 female CHWs with a minimum of 10 years of schooling and who reside in the same community participated in this formative study. The knowledge assessment of these CHWs before and after the training (Table 2) showed significant improvement (mean knowledge score before training: mean 8.00, SD 1.49; mean score post training: mean 11.40, SD 1.43; *P*<.001) after the training. In the posttraining qualitative feedback, CHWs showed confidence in their competency and community acceptance. They appreciated Sehat Ghar, especially its videos. One shared, “As soon as we play the video, the training gets refreshed in our mind. Also, the videos give us words to communicate better.” Some CHWs reported that their clients complained that the videos were lengthy. The CHWs improvised by narrating a brief story and going on to the discussion. One CHW shared:

*There are women who do not seem to have enough time. They are always busy with their children or other work and make excuses when we visit them. I do not show full videos in that case but tell them the main story. Eventually, I think, these women will develop interest.*

During the household visits observed for 20 families (Table 3), the CHWs showed high *fidelity to intervention*. They fulfilled (90% of visits) all the required steps except for praising the mother and family on accomplishments, which were observed during 58% of visits. The *mastery of using tablet* was very good to excellent, as all items received a high score (83%‐100%) in all the visits. Five items in *the completeness of intervention* received good to excellent scores (85%‐92%), while the other 5 received moderate to good (33%‐80%) scores. The items that did not receive an adequate score included using the main picture to build discussion, giving the mother enough time to speak, using the full session content, including videos, and reminding the mother about the practical tasks.

**Table 2.** Mean knowledge scores of community health worker before and after the training (N=10).

Question	Before, mean (SD)	After, mean (SD)	*P* values
Q1. How many ANC^a^ are required during a pregnancy?	0.50 (0.53)	0.9 (0.31)	.02
Q2. Most women take iron tablets during pregnancy; what is the benefit?	0.70 (0.49)	0.8 (0.42)	.17
Q3. Out of these who is a skilled birth attendant? (more than 1 options allowed)	0.70 (0.49)	1 (0)	.04
Q4. What should be given to a newborn as the first food?	0.80 (0.38)	0.9 (0.31)	.17
Q5. When should we bathe a baby first time after birth?	0.50 (0.53)	0.9 (0.31)	.05
Q6. For how long should a baby receive only mother’s milk?	0.70 (0.49)	0.9 (0.31)	.08
Q7. In summer, a 3-mo old baby can be given water with breastfeeding. True or false?	0.50 (0.53)	0.9 (0.31)	.02
Q8. How many times should a child receive vaccination until 15 mo of age?	0.30 (0.48)	0.6 (0.51)	.04
Q9. What effect will vaccination have on a child?	0.90 (0.31)	1 (0)	.17
Q10. A child starts learning immediately after birth.	0.20 (0.42)	0.8 (0.42)	.002
Q11. Special toys are necessary in order to play with a child.	0.80 (0.42)	0.9 (0.31)	.17
Q12. Who is responsible for a child’s good upbringing?	0.60 (0.52)	0.8 (0.42)	.08
Q13. Home environment has an effect on a child’s physical and cognitive development.	0.80 (0.42)	1 (0)	.08
Total	8.00 (1.49)	11.40 (1.43)	.001

a ANC: antenatal checkup.

**Table 3.** Community health worker’s performance in engaging with family and using the tablet and application during the 20 visits.^a^

Observation		Yes, n (%)	No, n (%)
**Fidelity**			
	1. Greets the family	20 (100)	0 (0)
	2. Has a friendly interaction with mother/other family members	20 (100)	0 (0)
	3. Asks questions about everyday routine to build the conversation	18 (90)	2 (10)
	4. Talks about previous visit, listens to what mother has to say	18 (90)	2 (8)
	5. Praises the mother/family over tasks they have done.	12 (58)	8 (42)
	6. Gives satisfactory answers to mother/family’s questions before moving on to the new content	18 (92)	2 (8)
**Mastery of using the tablet**			
	7. Can lock/unlock the gadget without difficulty	20 (100)	0 (0)
	8. Is confident about swiping between different screens while using the application	10 (83)	2 (17)
	9. Knows all the key functions well and when to use them. (volume/power/back key)	17 (83)	3 (17)
	10. If the device gets locked while delivering the visit, knows how to turn it back on.	20 (100)	0 (0)
	11. Takes care of the protection of the gadget while using it.	20 (100)	0 (0)
**Command over the content**			
	12. Picks the appropriate visit according to month of pregnancy/age of child.	18 (92)	2 (8)
	13. Uses the main picture to build a discussion around the topic	4 (20)	16 (80)
	14. Asks the mother’s views first regarding the behavior of focus	12 (58)	8 (42)
	15. Pauses in between the videos, asks mother about her opinion	17 (85)	3 (15)
	16. Discusses the problem and its potential solution	18 (92)	2 (8)
	17. Shows complete video	13 (67)	7 (33)
	18. Shows both the videos	18 (92)	2 (8)
	19. Is fluent with the content	17 (85)	3 (15)
	20. Talks about all the pictorial action points	13 (67)	7 (33)
	21. Reminds the mother about practical tasks, marks them on the calendar.	13 (67)	7 (33)

a Scoring: excellent >90%, very good 80%‐89%, good 70%‐79%, need improvement <69%.

In a typical visit, the CHWs spent 45‐60 minutes with the mother and her family. Mothers and their families were happy to have the CHW visit them and discuss the mother and baby’s health. “Nobody has ever come to visit us as these women do, and no one even comes to give us information verbally,” said one mother. Mothers appreciated the idea of conveying helpful information through videos and wanted to receive it consistently. They said it increased their knowledge and changed some of their practices. One shared:

*There were many things we did not know or were doing wrong. I was taking iron tablets with milk, learned the correct method through these visits, and now I take them with water.*

The study team and the CHWs also faced a few difficulties. The absence of a public sector health care system in the study areas narrowed the chances for the team to access the households. To open doors, we engaged 15 health workers but had to work with 10 from start to end, as the rest had to drop out for the aforementioned reasons. Internet connections, required mainly to download the application updates, were sporadic in some field areas. The study team, therefore, timed all the Sehat Ghar updates with monthly meetings the CHWs held at the main office where high-speed internet was available. The length of the videos was a challenge for a few mothers because they had to balance watching the videos with domestic responsibilities. However, the majority were interested in the characters and stories presented in the videos and watched them from start to end.

## Discussion

In this study, we developed a digital intervention that volunteer CHWs could use to deliver health education with basic training and some supervision. The exploratory part of this study revealed that families living in underserved areas unattended by PHC services were unaware of basic health promotion and disease prevention. Women relied on traditional birth attendants for antenatal checkups and went to the hospital only at the time of delivery, with no or minimal postnatal checkups. Newborn care was marginal as was the knowledge about a growing child’s health and nutrition. The home environment offered mixed conditions for child development. With the help of minimally trained volunteers from the community, mothers received infotainment-based health education. The volunteers and their audience mothers liked the home visits and the content of the intervention and wished to engage in this activity regularly.

Using videos as a tool to deliver counseling sessions had several advantages. First, the story format was interesting both for the mother and the health worker. Moreover, the characters (shown in an environment similar to the participant family) were taking actions about which health workers would ask questions warranting the mother’s attention. Second, the same videos were being used by all health workers ensuring adequate dose and fidelity [32]. This complete and acceptable delivery of interventions has been reported as a challenge when print materials are used along with the oral delivery of the content [33]. Third, the same videos could be used by thousands of lady health workers of the national program [34] after successful proof of effectiveness.

In contrast to some of the published studies in which such sessions turned into a passive watching of the video [35], our intervention used CBT-based principles that provided a structure to the session, with the video acting as a supporting tool. This meant that both the health worker and the mother must actively watch the video in anticipation of the questions that the health worker would ask, and the mother would respond about the situations faced by the characters in the video. Each discussion finished with both the health worker and the mother or other family members discussing the relevance of the content with their situation, identifying their problems, and working on joint solutions as reported in earlier studies [36].

Technology-based interventions like SMS text message reminders via mobile phones, robocalls, and social media have been used in the past, with their top-down communication and delivery from a distance limiting their effectiveness [37,38]. Studies assessing other behavior change strategies, like messaging campaigns through mass media, have found that they may not be effective when used in isolation [39]. Combining media and technology with face-to-face communication works better [40]. However, health content delivered through face-to-face communication by workers trained in a cascade setting dilutes the intervention with each step of trickle-down and must be addressed by a “cascade-plus” approach [41]. We embedded our digital application in a cascade-plus training of CHWs in which they were provided more than one job aid and multiple learning opportunities to discuss and improve their skills.

Digital interventions for CHW programs have received criticism because of their costs, the need for internet coverage, and intense training requirements [12]. However, our experience was different. We spent a total of US $4000 on this digital intervention—a one-time cost on an application and its videos that can be used by thousands of CHWs. Similarly, Android phones (US $100 in the local market) are owned by a large majority in the country and this application can be adapted for mobile phones. The common availability of smartphones helped in training the CHWs because most of them already knew the operating functions of the device. The lack of high-speed internet in the community did not pose a substantial challenge as the Sehat Ghar application was not dependent on installing updates while in the field or uploading data from the field.

In addition to videos, the identification and training of volunteer women and preparing them for the basic CHW role is also a critical element of our intervention. Such volunteers can be particularly relevant to settings where women have nominal education, families are resource poor, and deeply embedded cultural practices impede the adoption of healthful behaviors. In a community without the coverage of PHC, we were able to identify and train health volunteers who learned using a video-based digital application and then provided health education to mothers and families to facilitate a positive change in their health behaviors. This strategy can be useful in emergency and disaster situations, where infrastructure is badly damaged and essential health services have to be restored quickly.

Though this study was conducted before the pandemic, it has some insights applicable to outbreak situations as well. The video-based health content could be uploaded to other media for a consumer interested in watching stories to learn about her health. The same could be done by adding video content on the prevention of COVID-19 for an internet user (or a health worker) to download amid the lockdowns. Two similar “distance-compliant” strategic communications in Pakistan (ie, COVID-19 prevention messages used as caller ringtones on mobile phones, and the 24-hour national call-in helpline 1166) proved to be highly effective during the pandemic [42]. This implies that a digital health app already present on a mobile phone can be quickly updated with new information, including videos, in outbreak situations.

Some limitations are worth mentioning for a better interpretation of our findings. We wanted to conduct as many interviews and FGDs as required to reach a theoretical saturation point but had to limit our sample given the access issues. The high improvement in posttraining knowledge scores could be due to a smaller set of trainees who received good attention from the trainers. Moreover, they had less health knowledge previously, which could improve quickly. However, a change in knowledge does not guarantee a change in behaviors—the change in CHW practices observed over time will be a more meaningful outcome. The observations too have their limitation because of social desirability bias. However, we conducted these observations as part of “supportive supervision” in which the CHW knew that she would be helped and not scolded if she did not remember something and asked about it.

While we look forward to the implementation of Sehat Ghar among the 250 families to provide important insights for upscaling, we also envisage a few challenges for large-scale real-world implementation. Adopting a new approach of counseling that we developed using CBT principles may be resisted because the countrywide lady health worker program will have to plan new training. Moreover, after adoption, these visits will need monitoring and supportive supervision across the country to help the workers and avoid passive use of the videos, as reported by other studies [36]. However, the application’s development was a one-time cost, and the application or only the videos can be used after downloading on thousands of digital devices.

Based on the results from this pilot phase, we concluded that developing an easy-to-use digital application like Sehat Ghar is possible with minimal resources. Its use by minimally trained volunteers and acceptance by the community make it feasible. Its systematic rollout can contribute to improving health behaviors, including health care use. Mothers appreciate interesting content like video stories and are willing to incorporate the information into their daily practices. Lastly, applying participatory approaches during the formative phase of such interventions is critical for their long-term adoption by the community and CHW programs.

## Abbreviations

CBT: cognitive behavioral technique
CHW: community health worker
FGD: focus group discussion
HCP: health care provider
IDI: in-depth interview
PHC: primary health care
